# Antireflection Enhancement by Composite Nanoporous Zeolite 3A–Carbon Thin Film

**DOI:** 10.3390/nano9111641

**Published:** 2019-11-19

**Authors:** Maksym Stetsenko, Salvatore A. Pullano, Tetiana Margitych, Lidia Maksimenko, Ali Hassan, Serhii Kryvyi, Rui Hu, Chun Huang, Roman Ziniuk, Sergii Golovynskyi, Ivan Babichuk, Βaikui Li, Junle Qu, Antonino S. Fiorillo

**Affiliations:** 1 Key Laboratory of Optoelectronic Devices and Systems of Ministry of Education and Guangdong Province, College of Physics and Optoelectronic Engineering, Shenzhen University, Shenzhen 518060, China; stetsenkomax@gmail.com (M.S.); 15alirao@gmail.com (A.H.);; 2 V. Lashkaryov Institute of Semiconductor Physics, National Academy of Sciences of Ukraine, 03680 Kyiv, Ukraine; maximenko_lida@ukr.net (L.M.); serkriviy@mail.ru (S.K.); babichuk@isp.kiev.ua (I.B.); 3 Department of Health Sciences, Magna Græcia University of Catanzaro, 88100 Catanzaro, Italy; pullano@unicz.it (S.A.P.); nino@unicz.it (A.S.F.); 4 Kiev Institute for Nuclear Research, National Academy of Sciences of Ukraine, 03680 Kyiv, Ukraine; margtanya@gmail.com; 5 Institute of Physics, Polish Academy of Sciences, 02-668 Warsaw, Poland; 6 Intelligent Manufacturing Division, Wuyi University, Jiangmen 529020, China

**Keywords:** zeolite 3A, carbon, antireflective coating

## Abstract

A straightforward and effective spin-coating technique at 120 °C was investigated for the deposition of a thin nanoporous layer with antireflection properties onto glass and indium tin oxide (ITO) coated glass. A mixture of zeolite 3A powder and high iodine value vegetable oil was deposited, creating a carbonic paste with embedded nanoporous grains. Experimental results evidenced excellent broadband antireflection over the visible-near-infrared wavelength range (450–850 nm), with a diffuse reflectance value of 1.67% and 1.79%. Structural and optical characteristics stabilized over time. The results are promising for the accessible and cost-effective fabrication of an antireflective surface for optoelectronic devices.

## 1. Introduction

Antireflective (AR) coating fabrication is a high-interest topic, intensively investigated in industry (e.g., solar cells) for improving light transmission and reducing reflection. Single and multi-layer dielectric-based structures, nanocomposites and metal nanoparticles, were investigated for achieving and optimizing AR properties [1]. One of the most promising techniques involves variation in the light refraction (lowering of refraction index) along the thickness by deposition of nanostructured layers, as in the case of porous or patterned surfaces. Hybrid composites with improved mechanical, electrical, and optical characteristics are well-designed materials with surface roughness. They can interfere with the incident light, creating a refractive index gradient, allowing the progressive bending of light rays, which, in turn, results in improved AR characteristics [2,3,4].

Among nanoporous materials, synthetic zeolite is a crystalline aluminosilicate solid with a well-defined and stable porosity [5]. Previous investigations of beta polymorph A and mordenite-framework-inverted zeolite have shown a reduction in reflected light to less than 1% in the visible spectral range [6]. Outstanding AR characteristics were obtained by properly fabricating zeolite thin film for optical lens and glass replacing the existing analogs [7,8,9]. 

A fast, straightforward, and cost-effective thin film deposition technique was investigated on zeolite 3A by Fiorillo et al. [10,11]. The process allows for the control over thickness, roughness, and geometry by spin-coating of high iodine value vegetable-oil/zeolite mixture and a subsequent annealing step, resulting in a thin zeolite film (e.g., from a few micrometers to one hundred micrometers depending on the process). The typical morphology and energy dispersive analysis of X-rays (ΕDX) have been investigated [10,11,12]. AR and optical-polarization properties for the zeolite 3A with 100 μm thickness and zeolite/Au composite film deposited onto (111) p-type silicon using modulation polarimetry have also been investigated [12,13,14,15]. 

Recently, a new class of material with unique fluorescence properties, such as carbon dots (C-dots) in zeolite, was investigated [16]. Synthesis of C-dots on the surface of NaY zeolite (solid host) was also reported [17]. It was shown that thickness of AR coatings is significantly greater than the light wavelength. Therefore, a 3D forest-like structure made of carbon nanotube provides a highly effective technique for light trapping [18,19]. Thus, such a textured surface (ordered and disordered) with a few-micrometer thickness, was used to randomize the angular scattering and enhance the effective path length for light absorption and the AR effect [20].

In order to achieve broadband AR coatings for solar cells, a straightforward and scalable process is desirable [21,22,23,24]. Thus, the proposed deposition technique is highly promising because of easy transferal to mass production, cost-effectiveness, and compatibility with integrated circuit technology.

Herein, we reported an alternative way for low-cost fabrication of AR coatings with improved AR characteristics on a glass substrate with zeolite 3A–carbon film on top. In the study, dielectric and conductive substrates were investigated for a possible translational application in solar cell fabrication. Structural and AR characterizations were carried out by means of scanning electron microscopy (SEM), X-ray diffraction (XRD), excitation-emission spectroscopy, luminescence microscopy imaging, and Raman, visible-near-infrared (Vis-NIR), and modulation-polarization spectroscopy (MPS).

## 2. Materials and Methods 

### 2.1. Preparation of Substrates

Glass slides of 10 × 15 mm^2^ (Schott AG, Mainz, Germany) were used as substrate, being cleaned in acetone and isopropyl alcohol, and then sonicated for 5 min and dried in a N_2_ stream (refractive index of 1.52 (BK-7)). The sample was then placed into a piranha solution (H_2_SO_4_/H_2_O_2_, 2.5:1 (*v*/*v*)) for 30 min at room temperature; then, washed with deionized water and dried in the N_2_ stream to remove surface contaminants. A 100 nm layer of indium tin oxide nanofilm (ITO) was deposited onto the glass as a transparent conductive layer (Diamond Coatings, Halesowen, UK); see Figure S1. 

### 2.2. Materials

Zeolite film was deposited using a spin-coating process at a temperature of 120 °C for 3 h [10,11]. The mixture was obtained by dispersing a fine-grained powder of zeolite 3A (1–5 μm) [11] in soybean oil (with a molar ratio of 70:30 *w*/*w*) using an Ultra Turrax (IKA, Staufen, Germany) disperser at 25,000 rpm. The mixture was then deposited on one side of the substrate by spin-coating at 4000 rpm for 60 s, and thereafter annealed at 120 °C for 3 h. This resulted in a 10 μm thick layer consisting of the zeolite held together by a carbon paste obtained by the oil decomposition, which acted as a supporting matrix for the nanoporous crystals in contact with the substrate. This process occurred at a relatively low curing temperature, while the choice of oils to be used was made according to the number of double carbon bonds (the more the better) or equivalently, a higher iodine value, as previously investigated [10,11].

### 2.3. Materials Characterizations

SEM analysis of the porous zeolite 3A layer was performed using an EVO HD15 microscope (Carl Zeiss, Oberkochen, Germany). The thickness, evaluated using a profilometer Dektak 6M (Veeco, Plainview, NY, USA), was 10 μm, with the highest roughness being 1 μm. The mean value roughness in the sub-micron range (200–400 nm) was registered at the surface along a 500 µm distance.

XRD characterization of the structural properties and phase composition was carried out by means of a X’Pert Pro MRD XL (PANalytical, Malvern, UK) equipped with a Cu_Kα_ source of radiation (*λ* = 0.15406 nm). A W/Si parabolic mirror was used to create a high intensity parallel beam. The diffracted beam was collimated by a parallel plate collimator with the acceptance angle 0.27° used in combination with a 0.04 rad Soller slit. 

The photoluminescence excitation and emission spectra were carried out at room temperature using a Fluorolog i320R spectrofluorometer (Horiba, Kyoto, Japan), equipped with a 450 W xenon lamp and a Si CCD detector FL-1073 (Horiba). The nanosecond fluorescence decays were measured by using the time-correlated, single-photon counting (TCSPC) technique, a Fluorolog i320R spectrofluorometer, and a Horiba 370 nm NanoLED.

The visible fluorescence micrographs were obtained at room temperature, employing a Nikon Eclipse Ti-U inverted microscope (Nikon, Tokyo, Japan) at 20X optical magnification equipped with a Nikon DS-Fi2 digital camera. A Nikon light source was used for the excitation. The images were captured at UV, blue, or green light excitations through band-pass filters cubes: (i) Excitation (Ex.) 375/28 nm, Dichroic mirror (D.m.) 405 nm, Emission (Em.) 460/60 nm; (ii) Ex. 480/30 nm, D.m. 505 nm, Em. 535/45 nm; (iii) Ex. 540/25 nm, D.m. 565 nm, Em. 605/65 nm. For the imaging in the entire visible spectrum, the UV excitation and a long-pass filter were used (see Figure S2 in Supplementary Material). 

An inverted confocal Raman Alpha300Ri spectrometer was used for Raman spectroscopy at 532 nm excitation using an Nd:YAG DPSS laser at power 45 mW. The laser radiation was focused on sample with a 50× objective of a confocal Olympus microscope to a spot of ∼1 μm in diameter. 

Extinction and specular reflection measurements were carried out using a single beam spectrometer, UV–Vis SpectraScan (ScanSci, Vila Nova de Gaia, Portugal). Specular reflection spectra, also known as regular reflection, were recorded at an incidence angle of 45° for unpolarized light. Extinction spectra were recorded in transmission geometry at light normal incidence. The extinction is defined as the removed optical power from an incident beam. This power is the sum of the absorbed and scattered electromagnetic energy [25]. Diffuse reflectance spectra at room temperature were recorded using a double-beam Lambda 35 UV–Vis spectrophotometer (Perkin Elmer, Waltham, Massachusetts, USA) with an integrated 50 mm sphere (Labsphere RSA-PE-20). Optical characterization of the zeolite coatings was carried out employing modulation polarimetry. The Q (*ρ*(*λ*) = *R_s_* − *R_p_*) and V-components of the Stokes vector were measured in external reflection geometry at an incidence angle of 45° [26]. The external spectra of reflectance for s-polarized and p-polarized light, *R_s_* and *R_p_*, respectively, were also measured [12].

## 3. Results and Discussion

### 3.1. SEM and XRD Analyses

**Figure 1:**
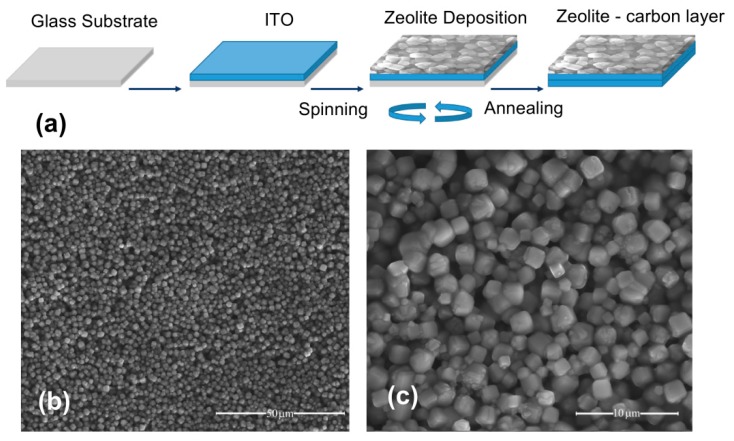
(**a**) Stages of the zeolite 3A deposition onto substrates. SEM surface morphologies for the samples deposited on (**b**) glass (scale bar 50 μm) and (**c**) ITO/glass (scale bar 10 μm).

Stages of the zeolite 3A deposition onto a glass surface and morphology of the zeolite coating surface are shown in Figure 1. Porous zeolite film (porosity of ~3 Å) is a grainy structure with particles 1–5 μm in size with cubic shapes and a good degree of adhesion.

**Figure 2:**
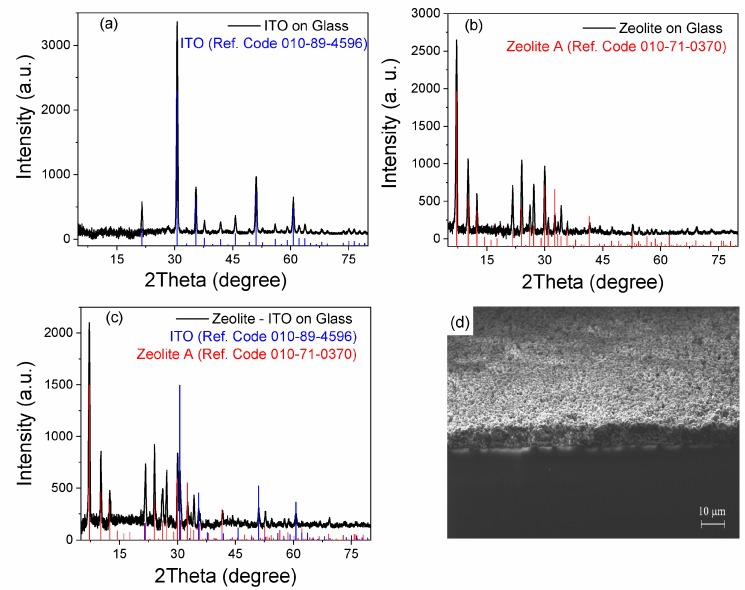
XRD patterns of (**a**) the ITO/glass, (**b**) zeolite/glass, and (**c**) zeolite/ITO/glass samples along with (**d**) an SEM cross-section of the zeolite/glass.

The X-ray diffractograms indicate a high crystallinity of the samples (Figure 2). Peaks typical for the zeolite A can be observed in both the samples [27]. The plots show all the characteristic peaks observed in a database for zeolite 3A with corresponding formulations (reference code 010-71-0370) and ITO (reference code 010-89-4596) [28]. XRD analysis does not show changes after one week and one-month, highlighting the stability of the layer.

The deposition technique implies the presence of carbon around the zeolite grains and possible incorporation into them [10]. The annealing process of soybean oil together with the zeolite 3A grains leads to the formation of supporting carbon matrix. The annealing under a similarly low temperature, led to the formation of soybean C-dots (SCDs) [29]. XRD patterns of our samples, Figure 2b,c, also displayed a broad peak centered at 25°, attributed to highly disordered carbon atoms [30,31].

### 3.2. Raman Spectroscopy 

**Figure 3:**
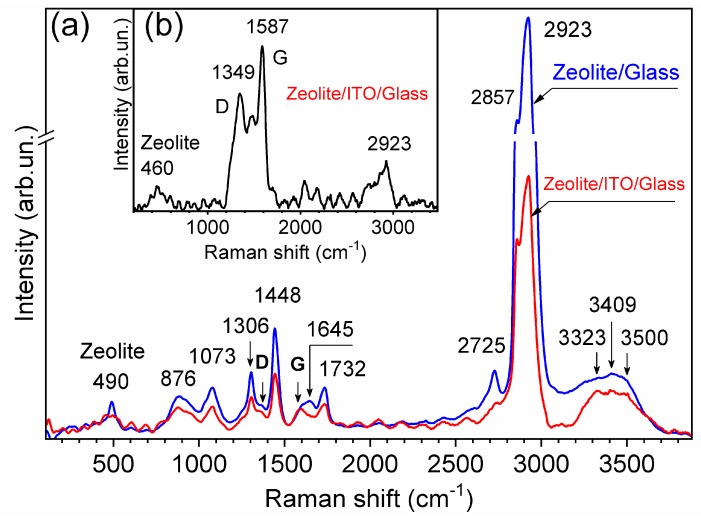
Raman spectra of the zeolite 3A—carbon layers (*λ_Ex_* = 532.5 nm): (**a**) laser power of 45 mW, (**b**) laser power—15 mW.

Raman spectroscopy is carried out to probe the graphitization and the crystallinity of carbon and C-dots in the zeolite 3A–carbon composite (Figure 3). The D (disorder) or G (crystalline) carbon bands with low amplitude were detected in the Raman spectra (*λ_Ex_* = 532 nm, laser power of 45 mW) [32,33]. The assignments of all Raman peaks appearing in the spectra are presented in Table 1. For most synthetic zeolites, the strongest band in the Raman spectra is observed in the region 450–600 cm^−1^. For zeolite A, T–O–T vibration mode is registered close to 489 cm^−1^ [34]. Small amplitude peaks at 689 cm^−1^ and 558 cm^−1^ are related to the presence of four and six-member double rings of zeolite, respectively [35].

The D-band located at 1346–1380 cm^−1^ corresponds to amorphous sp^3^ hybridized carbon, while the G-band at 1580–1600 cm^−1^ refers to the presence of a well-ordered sp^2^ hybridized graphite structure [33]. The D-band is related to the scattering processes induced by defects in the graphite structure. Therefore, the intensity ratio *R* = *I_D_*/*I_G_* reflects the graphitization degree.

The above-reported carbon Raman bands are also found for C-dots, where the G-band confirms the existence of the sp^2^ hybridized (crystalline) core, and the D-band accounts for defects and disordered carbon associated with amorphous carbon [41]. A small *I_D_*/*I_G_* ratio of ~0.5 designates that the carbonization process during the synthesis leads to a high-crystalline C-dots core, while larger ratios indicate a growing disorder and/or amount of amorphous carbon in C-dots [41,42]. Therefore, in order to confirm the presence of D and G-bands, we conducted additional measurements with a laser radiation power of 15 mW (Figure 3b). This allowed reducing the intensity of other vibrational bands and more accurately monitoring the D and G-bands. Consequently, we found that *I_D_*/*I_G_* was equal to 0.7. Thus, the resulted zeolite 3A–carbon layer is characterized by a large amount of disorder.

### 3.3. Antireflectance and Optical-Polarization Properties

**Figure 4:**
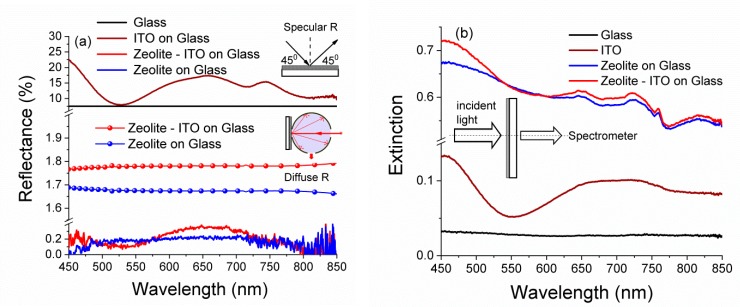
Spectra of (**a**) specular and diffuse reflectance and (**b**) extinction for the glass, nanofilms ITO, zeolite/glass, and zeolite/ITO/glass.

The spectral performance of the micro/nanotextured surfaces such as zeolite-carbon coatings were characterized through the measurements of specular reflectance, diffuse reflection, and extinction. The influence of substrate materials on AR properties of the zeolite coating was investigated via Vis-NIR reflectance and extinction. The results obtained for the AR coatings deposited on glass and ITO/glass are summarized in Figure 4.

With the aim of investigating the effect of the annealing process, ITO deposited on bare glass underwent through the same annealing process (at 120 °C for 3 h) simultaneously with the nanoporous coated substrate. 

The ITO reflection spectra for ITO-coated glass were found in good agreement with [17]. The average specular reflection coefficient over the range 450–850 nm was found to decrease from ~15% for the uncoated ITO/glass substrate down to 0.25% for the coated substrate. The diffuse reflection was about 1.79%. Note that the reflectance of the AR coated glass was always substantially lower than that the glass (a typical reflectivity of glass is ~8%) [7]. However, due to the deposition of zeolite 3A, the specular reflectance in broadband wavelength range was reduced down to 0.23%, and more importantly, the diffuse reflection was ~1.67% (Figure 4a). The spectral characteristics of specular reflectance were roughly estimated by averaging several measurements taken at different positions on the sample surface (Figure S3).

These results are promising when compared with others obtained by the sol-gel method for single-layer zeolite [7]. 

Diffuse reflectance was obtained due to a combination of the granular structure properties of zeolite-carbon coatings and the presence of carbon, as seen in Figure 4a. Carbon is one of materials used for antireflection coatings’ designs [43]. The disordered nanotextured carbon materials have broad absorption properties over the entire range from mid-IR to UV, as shown in [44]. Zeolite 3A-carbon layers evidenced a marked enhancement of the broadband absorption [21]. The extinction spectra for zeolite 3A-carbon coatings are characterized the sum of value absorbed and scattered light [25], as showed in Figure 4b.

The dense distribution of the zeolite 3A grains, combined with their size distribution and cubic shape with different orientations, contribute to their light trapping effect [20]. The morphology of zeolite 3 A–carbon layers lead to the incident light being reflected and refracted in layers and grains, eventually being effectively absorbed (Figure 4b).

**Figure 5:**
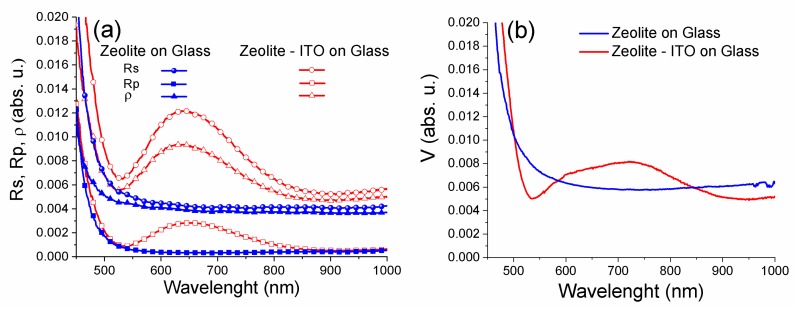
Polarization spectral characteristics of the zeolite 3A–carbon coatings: (**a**) amplitude and (**b**) phase anisotropy.

Finally, in order to thoroughly characterize the AR properties of our zeolite 3A coatings on different substrates, we evaluated both the unpolarized and polarized (with both s and p-polarized light) specular reflectance spectra. Spectral characteristics of the polarization differences *ρ* (Q-component of Stokes vector) and polarized (with both s- and p-polarized light) reflectance spectra at the incident angle of 45° are summarized in Figure 5a.

The spectral characteristics of ρ(λ), Rs(λ), and Rp(λ) for zeolite 3A-carbon layers are characterized by the amplitude anisotropy of reflected light in the broad wavelength range. The same amplitude values for spectral characteristics of the V-component of the Stokes vector measured for registration circular anisotropy at the incident angle of 45° are shown in Figure 5b [45]. Moreover, for the same incidence angle and in the presence of polarized light, the specular reflectance is almost polarization-insensitive [46].

### 3.4. Fluorescence Spectroscopy and Imaging 

Excitation-dependent fluorescence spectroscopy was productively used for the investigation of carbon nanomaterials and C-dots with zeolite [16,17,29,31]. Information on the behavior of its spectral characteristics might be useful for the development of prospective solar cell coatings based on zeolite 3A–carbon composite materials.

**Figure 6:**
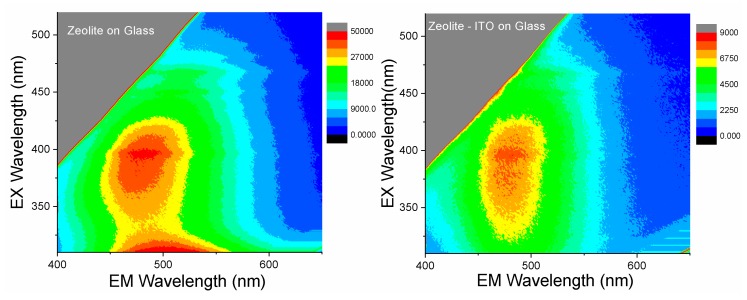
Excitation–emission two-dimensional plots for the zeolite 3A–carbon layers on glass and ITO/glass.

In Figure 6, fluorescence spectra of the zeolite 3A deposited onto the glass and glass covered with ITO nanofilm show broad emission bands in the 400–600 nm range. The presence of the ITO layer on the glass leads to a decrease in the intensity of the emission. Under UV excitation, the emission wavelengths remain the same, indicating that one fluorescent component dominates the emission typical for carbon nanomaterials close to 450 nm [47,48]. When exciting at 450–550 nm, the spectra red-shift and the intensity decreases gradually, suggesting the presence of different types of fluorophores having different degrees of double bond conjugation, and hence, different emission wavelengths [17,47,48]. This phenomenon has been widely observed in luminescent carbon nanomaterials. Also, in the case of SCDs when the excitation was beyond 330 nm, the emission was found to red-shift with the intensity, which gradually decreased with the increment of excitation wavelength, indicating different surface defects of SCDs [49]. Thus, the deposited zeolite 3A–carbon composite shows the combined properties in fluorescence spectra of different types of carbon materials, SCDs, and organic products of soybean oil (organic fluorophores) [29,50,51].

**Figure 7:**
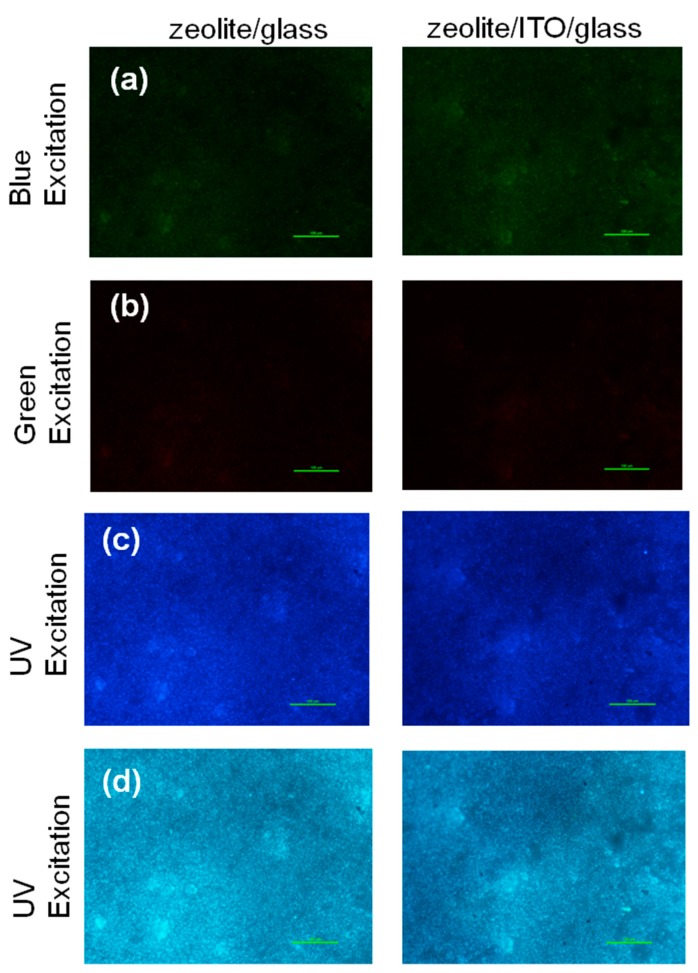
Fluorescence microscopy images of the zeolite/glass and zeolite/ITO/glass under blue (**a**), green (**b**) and UV excitations (**c**,**d**). The images (**a**–**c**) were obtained through the band-pass filters of different wavelengths; the images (**d**) were captured within visible range (scale bar—100μm).

In order to show visible, excited emission layered structures, fluorescence optical microphotographs for this material were captured at different excitation wavelengths and spectral ranges (Figure 7). The coating demonstrates emission within blue, green, and red ranges, when exciting with different wavelengths (Figure 7a–c). These images correlate with the fluorescence spectra. For emission the red spectral shift and reducing of intensity take place with the increasing of excitation wavelength [52]. Imaging in the entire visible region under UV excitation evidenced the zeolite 3A–carbon composite material shows near homogeneous blue color visible emission, suggesting a good distribution of emitting centers in the layers (Figure 7d). The observable emissions are one of the main traits of C-dots [27,53,54].

Porous zeolite frameworks with dopants are attracting much attending as novel porous materials for the construction of catalytic thin films coated on the conducting glass as a counter electrode to substitute costly platinum for quantum dot-sensitized solar cells [55]. Furthermore, C-dots and carbon materials have recently shown promise in this type of solar cell, playing the role of a cheap sensitizer replacement or functioning as a dopant in the photoactive material, electrolyte, or counter electrode of other device architectures (e.g., polymer, solid-state, and perovskite solar cells) [56]. Hence, the combination of the structural and optical properties of zeolite 3A–carbon nanomaterials is highly relevant for solar panel development.

### 3.5. Nanosecond Fluorescence Decay

**Figure 8:**
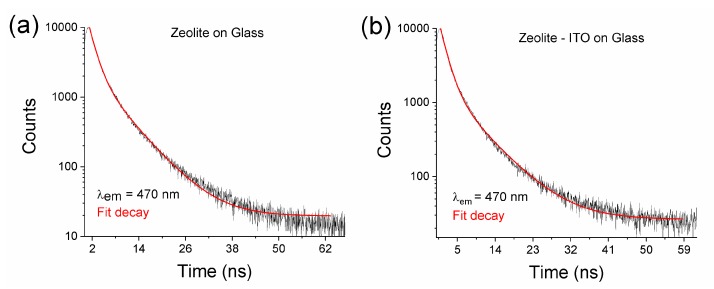
Fluorescence decays of (**a**) the zeolite/glass and (**b**) zeolite/ITO/glass (*λ_Ex_* = 370 nm, *λ_Em_* = 470 nm).

Under UV excitation at 370 nm, the composite layer emits blue light centered at ~470 nm (Figure 5). In order to obtain more insights about the fluorescence of zeolite, the decays were recorded at *λ_Em_* = 470 nm and *λ_Ex_* = 370 nm (Figure 8).

The time-resolved decay dependences reveal the presence of two components with lifetimes of *τ*_1_ = 1.69 (1.6) ns and *τ*_2_ = 6.63 (6.89) ns, respectively. This phenomenon should be directly linked to a high dispersion in size and shape of carbon nanomaterials, such as C-dots, due to the low temperature annealing process of zeolite 3A with soybean oil. The short lifetime of the zeolite 3A carbon is indicative of the radiative recombination of the excitons, giving rise to the fluorescence [57]. This also may be caused by the different chromophores or energy levels present in the samples and responsible for their multiple lifetimes [58]. 

Consequently, our AR coatings, based on structured zeolite 3A–carbon composite, showed luminescent properties with the effect of suppressing the light reflection. Moreover, the possibility of tuning refractive index by varying the pore size enables the fabrication of graded-index coatings also. The data we obtained are promising for designing an efficient photoanode for dye-sensitized solar cells [55,59]. 

## 4. Conclusions

The alternative, low-cost spin-coating technique at a low temperature (120 °C) was successfully used for the fabrication of zeolite 3A–carbon-based AR coatings. The coatings demonstrated the ability to reduce the light reflection from glass and electrode conducting material (ITO) with value equals to 1.67% and 1.79%, respectively. This feature makes the zeolite 3A layers more relevant as an AR coating in the Vis–NIR spectral range, due to its low-dielectric properties combined with absorbance and light trapping. We believe that the materials and techniques reported here are promising for the straightforward and cost-effective fabrication of AR surfaces for optoelectronic devices such as solar cell detectors.

## Figures and Tables

**Table 1:** Vibrational frequencies of the zeolite 3A–carbon.

Wavenumber, cm^−1^	Functional Group	Ref.
490 (460)	Zeolite A T-O-T	[34]
876	C–C str; CH2 rock	[36]
1073	Symmetric vibrations С-О-С, C-C, C- N	[37,38]
1306	γ-CH_2_	[39]
1349	D	[33]
1448	δ-CH_2_	[39]
1587	G	[33]
1645	-C–C-	[40]
1732	ν-C=O (ester)	[39]
2725	ν-CH (aliphatic –CHO)	[39]
2857	ν-CH (–CH_2_ symmetric)	[39]
2923	ν-CH (–CH_3_ symmetric)	[39]
3323, 3409, 3500	ν-CH (–CH_3_ symmetric)	[39]

## Supplementary Materials

The following are available online at https://www.mdpi.com/2079-4991/9/11/1641/s1. Figure S1: SEM ITO-glass cover slip. Figure S2: Optical parameters of band and longpass for fluorescence images. Figure S3: Spectral characteristics in different points of zeolite/ITO/glass coatings.
