# Supplementary material for: Antireflection Enhancement by Composite Nanoporous Zeolite 3A–Carbon Thin Film
Source: Nanomaterials (Basel). 2019 Nov 19;9(11):1641. doi: 10.3390/nano9111641 (PMC6915533; doi:10.3390/nano9111641)
Supplement: Supplementary file 1 [file nanomaterials-09-01641-s001.pdf]

nanomaterials

MDPI

Article

Antireflection Enhancement by Composite Nanoporous Zeolite 3A–Carbon Thin Film

Maksym Stetsenko [1,2], Salvatore A. Pullano [3], Tetiana Margitych [4], Lidia Maksimenko [2], Ali Hassan [1], Serhii Kryvyi [2,5], Rui Hu[1], Chun Huang [1], Roman Ziniuk [1], Sergii Golovynskyi [1,2], Ivan Babichuk [2,6], Baikui Li [1,*], Junle Qu [1,*] and Antonino S. Fiorillo [3]

1 Key Laboratory of Optoelectronic Devices and Systems of Ministry of Education and Guangdong Province, College of Physics and Optoelectronic Engineering, Shenzhen University, Shenzhen 518060, China; stetsenkomax@gmail.com (M.S.); 15alirao@gmail.com (A.H.); rhu@szu.edu.cn (R.H.); 2172281522@email.szu.edu.cn (C.H.); romanziniuk.94@hotmail.com (R.Z.); golovynskyi@gmail.com (S.G.)

2 V. Lashkaryov Institute of Semiconductor Physics, National Academy of Sciences of Ukraine, 03680 Kyiv, Ukraine; maximenko_lida@ukr.net (L.M.); serkriviy@mail.ru (S.K.); babichuk@isp.kiev.ua (I.B.)

3 Department of Health Sciences, Magna Græcia University of Catanzaro, 88100 Catanzaro, Italy; pullano@unicz.it (S.A.P.); nino@unicz.it (A.S.F.)

4 Kiev Institute for Nuclear Research, National Academy of Sciences of Ukraine, 03680 Kyiv, Ukraine; margtanya@gmail.com

5 Institute of Physics, Polish Academy of Sciences, 02-668 Warsaw, Poland; serkriviy@mail.ru

6 Intelligent Manufacturing Division, Wuyi University, Jiangmen 529020, China

* Correspondence: libk@szu.edu.cn (B.L.), jlqu@szu.edu.cn (J.Q.)

The SEM analysis was performed using EVO HD microscope from Carl Zeiss.

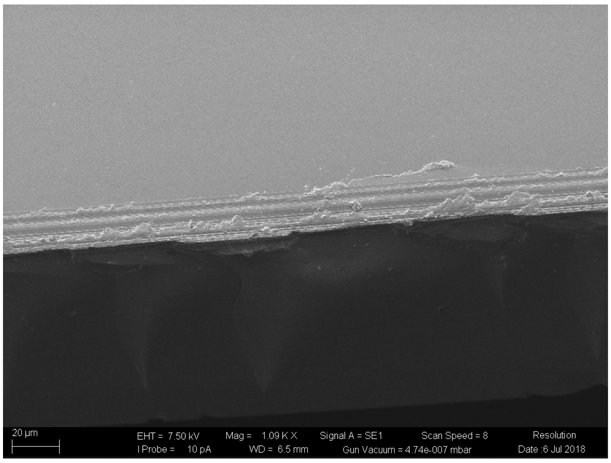

Figure S1 SEM ITO-glass cover slip

Bandpass filter cubes. Standard series

Filter Cube	Filter diameter	Excitation filter	Dichroic mirror	Barrier filter
**DAPI**	25 mm	375/28	415	460/60
**FITC**	25 mm	480/30	505	535/45
**TRITC**	25 mm	540/25	565	605/55

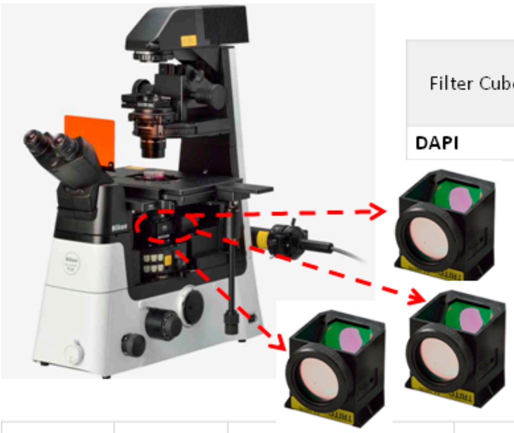

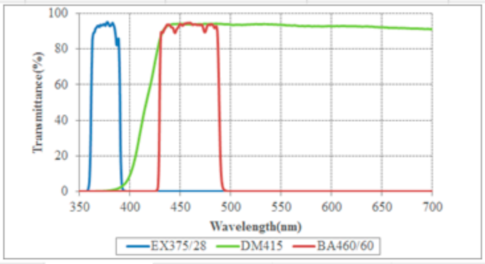

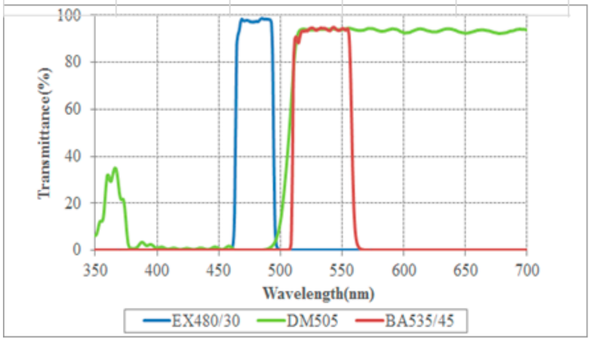

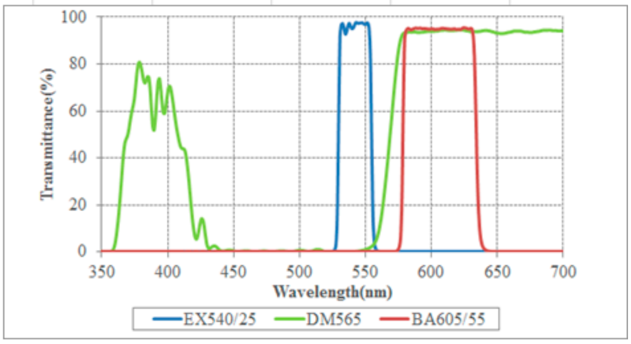

Longpass filter cubes

Filter Cube	Filter diameter	Excitation filter	Dichroic mirror	Barrier filter
	25 mm	375/28	415	420

Figure S2 Optical parameters of band and longpass for fluorescence images

The distance between the points at which the reflectance spectra were measured is 1 cm.

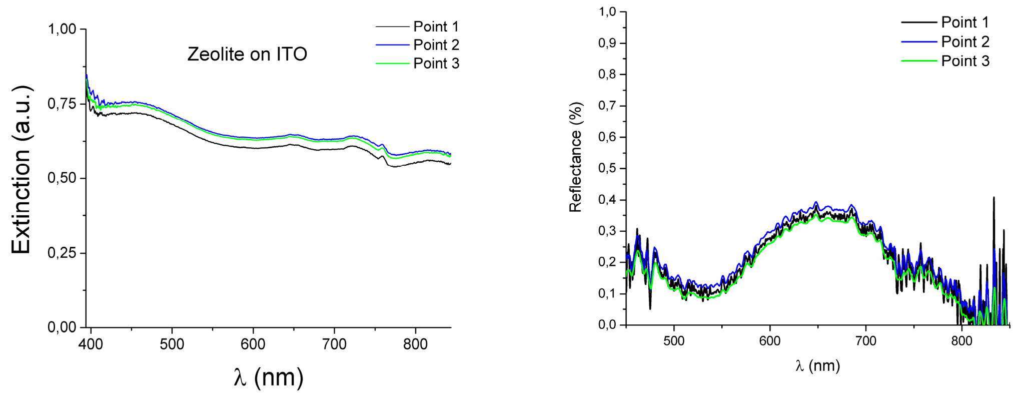

Figure S3 Spectral characteristics in different points of zeolite/ITO/glass coatings
